# Creating an electronic antibiogram using visualization software: Easily updatable and removes the need for yearly manual review

**DOI:** 10.1017/ash.2023.262

**Published:** 2023-09-29

**Authors:** Ashley Dauphin, Christopher McCoy, Robert Bowden, Matthew Lee, Howard Gold, Ryan Chapin

## Abstract

**Background:** Previously, our hospital manually built a static antibiogram from a surveillance system (VigiLanz) culture report. In 2019, a collaboration between the antimicrobial stewardship team (AST) and the infection control (IC) team set out to leverage data automation to create a dynamic antibiogram. The goal for the antibiogram was the ability to easily distribute and update for hospital staff, with the added ability to perform advanced tracking and surveillance of organism and drug susceptibilities for AST and IC. By having a readily available, accurate, and Clinical and Laboratory Standards Institute (CLSI)–compliant antibiogram, clinicians have the best available data on which to base their empiric antibiotic decisions. **Methods:** First, assessment of required access to hospital databases and selection of a visualization software (MS Power BI) was performed. Connecting SQL database feeds to Power BI enabled creation of a data model using DAX and M code to comply with the CLSI, generating the first isolate per patient per year. Once a visual antibiogram was created, it was validated against compiled antibiograms using data from the microbiology laboratory middleware (bioMerieux, Observa Integrated Data Management Software). This validation process uncovered some discrepancies between the 2 reference reports due to cascade reporting of susceptibilities. The Observa-derived data were used as the source of truth. The antibiogram prototype was presented to AST/IC members, microbiology laboratory leadership, and other stakeholders to assess functionality. **Results:** Following feedback and revisions by stakeholders, the new antibiogram was published on a hospital-wide digital platform (Fig. 1). Clinicians may view the antibiogram at any time on desktops from a firewall (or password)–protected intranet. The antibiogram view defaults to the current calendar year and users may interact with the antibiogram rows and columns without disrupting the integrity of the background databases or codes. Each year, simple refreshing of the Power BI antibiogram and changing of the calendar year allows us to easily and accurately update the antibiogram on the hospital-wide digital platform. **Conclusions:** This interdisciplinary collaboration resulted in a new dynamic, CLSI-compliant antibiogram with improved usability, increased visibility, and straightforward updating. In the future, a mobile version of the antibiogram may further enhance accessibility, bring more useful information to providers, and optimize AST/IC guidelines and education.

**Figure:**
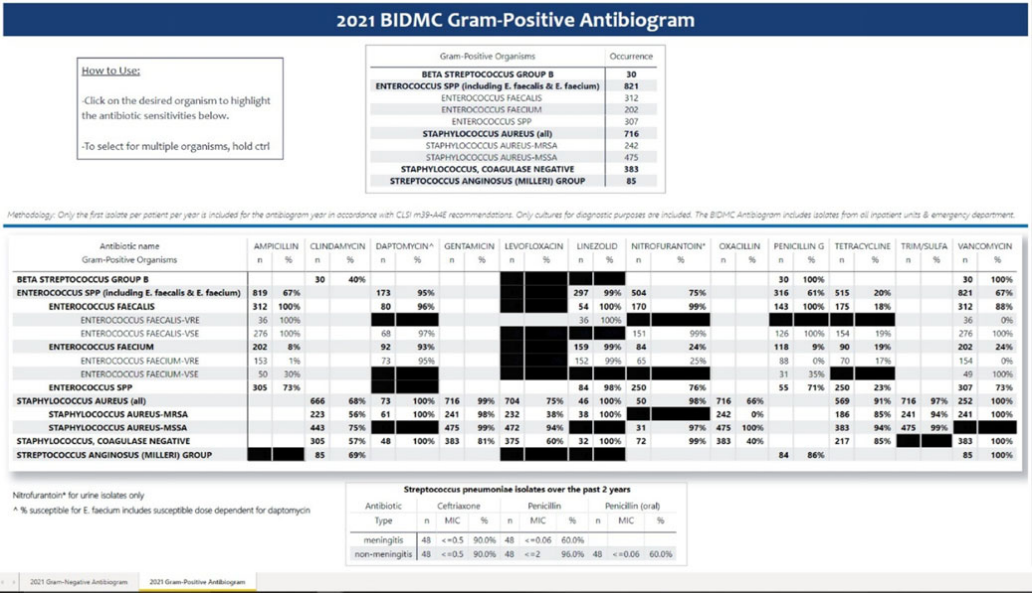

**Disclosures:** None

